# Chorioamnionitis and Lung Injury in Preterm Newborns

**DOI:** 10.1155/2013/890987

**Published:** 2013-01-10

**Authors:** Gustavo Rocha

**Affiliations:** Division of Neonatology, Department of Pediatrics, São João Hospital, 4202-451 Porto, Portugal

## Abstract

There is a strong evidence that histologic chorioamnionitis is associated with a reduction of incidence and severity of respiratory distress syndrome (RDS). Short-term maturational effects on the lungs of extremely premature infants seem to be, however, accompanied by a greater susceptibility of the lung, eventually contributing to an increased risk of bronchopulmonary dysplasia (BPD). Genetic susceptibility to BPD is an evolving area of research and several studies have directly related the risk of BPD to genomic variants. There is a substantial heterogeneity across the studies in the magnitude of the association between chorioamnionitis and BPD, and whether or not the association is statistically significant. Considerable variation is largely dependent on differences of inclusion and exclusion criteria, as well as on clinical and histopathological definitions. The presence of significant publication bias may exaggerate the magnitude of the association. Controlling for publication bias may conduct to adjusted results that are no longer significant. Recent studies generally seem to confirm the effect of chorioamnionitis on RDS incidence, while no effect on BPD is seen. Recent data suggest susceptibility for subsequent asthma to be increased on long-term followup. Additional research on this field is needed.

## 1. Introduction

Watterberg and colleagues [1] were the first to report, in 1996, a decrease in the incidence of respiratory distress syndrome (RDS), while the incidence of chronic lung injury, marked by the presence of bronchopulmonary dysplasia (BPD), was increased in preterm newborns with histological chorioamnionitis. This paradoxical effect of prenatal inflammation on pulmonary outcome has been referred to as the “Watterberg effect.” Since then, the effects of antenatal inflammation on both short- and long-term pulmonary outcome have received increasing attention, and many studies have been reported over the last years.

Chorioamnionitis is the leading cause of very preterm delivery and its incidence increases with decreasing gestational age [2–5].

BPD is one of the most frequent sequelae in very preterm infants and results in increased healthcare costs, prolonged hospital stays with frequent readmissions, and deleterious effects on subsequent growth and neurodevelopment [6, 7]. BPD is mostly multifactorial and a complex view of its pathogenesis (“multiple hits” hypothesis) has emerged, which includes antenatal exposure to a proinflammatory environment together with various postnatal inflammation-triggering events such as mechanical ventilation, sepsis, and patent ductus arteriosus [6, 8–10]. The “new” BPD results from an arrest of alveolar development with minimal fibrosis, associated with preterm birth, and is different from the “typical” BPD that follows severe RDS [6]. 

While the literature is consistent with the association between histological chorioamnionitis and a decreased incidence of RDS in the preterm newborn, many studies have found no association between chorioamnionitis and BPD [11, 12]. 

The purpose of this paper is to provide the actual evidence on neonatal pulmonary outcome after exposure to intrauterine inflammation.

## 2. Experimental Models of Inflammatory Neonatal Lung Injury

Animal models of intrauterine inflammation/infection provided evidence for altered lung development, with accelerated functional maturation and arrested alveolarization and vascular development. These models also demonstrated the central role of the developing immune system in the pathogenesis of lung injury.

In the sheep model [9, 13], sterile chorioamnionitis caused by a single injection of *Escherichia coli* lipopolysaccharide stimulated pulmonary inflammation with increased expression of proinflammatory mediators as cytokines, chemokines, monocyte chemoattractant protein, recruitment of polymorphonuclear cells and monocytes, maturation of monocytes to alveolar macrophages, increased secretion of surfactant proteins and phospholipids, and arrested alveolar and microvascular development. This experimental model demonstrated both, a maturational response with increased pulmonary compliance, as well as aberrant structural changes on the developing lung. Injection of *Escherichia coli* lipopolysaccharide into the amniotic fluid of E15 BALB/cJ mice [14] increased the luminal volume density of fetal mouse lungs at embryonic day (E) 17 and E18. Lipopolysaccharide also increased luminal volume and decreased distal lung branching in fetal mouse lung explants. This effect required NF-kappaB activation and functional Toll-Like receptor 4. Airway branching may require fibronectin-dependent epithelial-mesenchymal interactions, representing a potential target for innate immune signaling. Antifibronectin antibodies and lipopolysaccharide both blocked distal lung branching. By immunofluorescence, fibronectin localized to the clefts between newly formed airways but was restricted to peripheral mesenchymal cells in lipopolysaccharide-exposed explants. These data suggest that lipopolysaccharide may alter the expression pattern of mesenchymal fibronectin, potentially disrupting epithelial-mesenchymal interactions and inhibiting distal airway branching and alveolarization. This mechanism may link innate immune signaling with defects in structural development of the fetal lung.

Two studies [15, 16] on a bitransgenic mouse model revealed that human cytokine-1 beta expression in the saccular stage was sufficient to cause a BPD-like illness in infant mice, whereas the lung was more resistant to cytokine-1 beta induced injury at later developmental stages. This is consistent with the clinical observation that extreme prematures (23–27 weeks, early saccular stage) are at the highest risk for inflammation-mediated BPD, whereas infants over 32 weeks (late saccular-alveolar stage) are at much lower risk.

## 3. Experimental Models of *Ureaplasma* Intrauterine Infection

The genital mycoplasmas *Ureaplasma urealyticum* and *Ureaplasma parvum* are the most common organisms isolated in the amniotic fluid in women with preterm labor and the rate of vertical transmission increases with the duration of membrane rupture [17, 18]. Although the ascending infection at or near the time of delivery has been pointed as the most common route of infection, *Ureaplasmas* have been detected in 13% of amniotic fluid samples at the time of genetic amniocentesis, at 16–20 weeks gestation, in asymptomatic women, indicating possible prolonged subclinical infection [17]. There is now considerable clinical and experimental evidence that these organisms contribute to chorioamnionitis, fetal inflammatory response, preterm birth, and neonatal morbidities including BPD, intraventricular hemorrhage, and necrotizing enterocolitis [13, 17, 19–28]. Pathologic changes in *Ureaplasma*-infected lungs of preterm infants are characterized by moderate-severe fibrosis, disordered elastin accumulation, myofibroblast accumulation, and chronic inflammation [29, 30]. 

Intrauterine *Ureaplasma* infection models in nonhuman primates [22, 26, 31], sheep [13], and mice [32] closely mimic the human exposure during early stages of lung development. In all intrauterine models, *Ureaplasma* organisms established a persistent infection in the intrauterine compartment, indicating limited capacity to clear these organisms. The findings of these studies suggest that *Ureaplasma* infection causes an imbalance of proinflammatory, profibrotic, and anti-inflammatory, antifibrotic factors in the fetal lung that may be augmented by postnatal exposure to hyperoxia and mechanical ventilation. 

Humans are the specific host for *Ureaplasma parvum* and *Ureaplasma urealyticum*, so the organisms may elicit a less robust response in less related species. Alternatively, as shown in the mouse model, acute inflammation occurring during the saccular stage but not other stages of lung development results in arrested alveolarization, and airway remodelling typical of human BPD.

## 4. Genetic Susceptibility

Several studies have directly related the risk of BPD to genomic variants [33]. Polymorphisms of cytokines (IFN*γ* T^+874^A), adhesion molecules (L-selectin-Prot213Ser), elements of rennin-angiotensin system (ACE-I/D), antioxidant enzymes (GST-P1 Val105lle), and surfactant proteins (SPA1, SPB intron4) have been identified as risk factors for BPD. Other studies investigated the role of genotype in BPD risk factors. Premature birth has been linked to polymorphisms with an impact on immune status (such as IL-6 G^−174^C, MBL2 54G/A, VEGF G^+405^C, and HSP72A^+1267^G genes) and matrix metalloproteases. Fetal inflammatory response syndrome, a major determinant of BPD, is also affected by genotype (including LT*α*A^+250^G) [33]. 

In our pilot study [34], HLA-A*68, -B*51, and -Cw*14 were the human major histocompatibility complex alleles associated with oxygen requirement at 36 weeks post-menstrual age in preterm neonates less than 32 weeks gestational age. The idea that an autoimmune process might be involved in BPD pathogenesis is novel and needs further investigation.

Twin concordance studies have suggested that the contribution of genetic risk to BPD is high, accounting for 35%–65% risk for the outcome [35, 36]. 

The challenges of enrolling adequate sample size in the preterm population, racial/ethnic heterogeneity in populations, variations in clinical practices, and the multifactorial pathogenesis of BPD make genetic studies difficult to be performed and may be responsible for the fact that some associations have not been replicated in subsequent studies.

## 5. Chorioamnionitis and Neonatal Respiratory Outcome in Preterm Infants

The primary process in the aetiology of intrauterine inflammation is believed to be ascending bacterial invasion from the cervicovaginal tract, although several other routes have been postulated [2, 37]. Extrauterine infections such as periodontitis, pneumonia, and urinary tract infections are risk factors for preterm birth [38, 39]. In the rodent model, maternal systemic inflammation resulted in prolonged pulmonary inflammation postnatally and a BPD phenotype [39]. 

Clinical chorioamnionitis does not correlate well with histological chorioamnionitis or culture positive amniotic fluid [40]. Histopathological examination of the placenta is the gold standard for evaluating antenatal inflammatory processes that might influence fetal development. Histological chorioamnionitis develops through a well-characterized stereotyped progression of maternal and fetal cellular stages that vary from patient to patient and are amenable to quantification. Increases in the intensity of these responses and their gradual transformation into a chronic phase are important variables that can adversely affect fetal physiology. Under recognised placental inflammatory lesions affecting the decidua, placental villi and fetal vessels are also potentially informative factors that should be taken into account in the studies of adverse pregnancy outcomes [37]. 

Most deliveries before 30 weeks gestation (saccular stage of lung development) are associated with histological chorioamnionitis, which is often clinically silent [2]. The more preterm the delivery, the more often the histological chorioamnionitis is detected [3]. Most infants delivered before 30 weeks gestation also have amniotic fluid that is culture positive for low pathogenic organisms such as *Ureaplasma* and *Mycoplasma* [2]. 

Lung inflammation is defined as increased inflammatory cells in the airspaces and lung tissue that are producing proinflammatory mediators such as hydrogen peroxide, interleukin 1, and interleukin 8 [40]. Lung inflammation starting before delivery with chorioamnionitis may continue as a result of routine care practices (ventilation, oxygen exposure), and adverse clinical events, such as nosocomial infection. The proinflammation is counterbalanced by anti-inflammatory effects of antenatal steroids, because about 80% or preterm infants are exposed to betamethasone or dexamethasone [40]. Postnatal steroids also acutely decrease indicators of inflammation. Both antenatal and postnatal steroids inhibit alveolar septation. Both proinflammatory and anti-inflammatory mediators disrupt alveolarization and to date no treatments are available to promote alveolarization [40]. 

Histological chorioamnionitis is defined by a maternal inflammatory response with neutrophilic infiltration of the membranes and/or chorionic plate. Fetal inflammation has been defined as either chorionic vasculitis [41], umbilical vasculitis [4], funisitis [42–45], “fetal response” [46], or subdivided into polymorphonuclear leukocyte infiltration of the chorionic plate or the umbilical cord [47]. Some studies showed that the RDS incidence was further decreased in infants with fetal involvement when compared to those with only maternal signs of inflammation [4, 41, 45, 47], an effect that appears to be additive to that of chorioamnionitis alone [4]. None of the studies found fetal inflammation to increase the risk of developing BPD, when compared to only maternal inflammation.

The studies where the association between chorioamnionitis and RDS has been assessed presented either similar [34, 43, 45, 48–52] or decreased [1, 4, 5, 47, 53, 54] RDS incidences. Today, it is generally accepted that there is enough evidence to consider that histological chorioamnionitis is associated with a reduction of incidence and severity of RDS [11, 12, 55].

Data on the need for respiratory support after chorioamnionitis differ greatly between studies. Often parallel to RDS incidence, chorioamnionitis has been reported to increase [49, 52] and decrease [53], as well not to affect the need for surfactant administration [42]. Moreover, while some report no effect on the need for mechanical ventilation [52, 53], time spent on the ventilator [48], and time on additional oxygen supplementation [48], others report increased need for ventilatory support or oxygen [51, 52].

The association between chorioamnionitis and BPD has been assessed in several studies, yielding inconsistent results. In order to clarify this issue, Hartling and colleagues [12] conducted a systematic review including an extensive and comprehensive search, duplicate screening, inclusion and data extraction to reduce bias, and metaregression to control for potential confounders. The authors identified 3,587 potentially relevant studies, of which 59 (15,295 patients) met the inclusion criteria. Studies were included if they had a comparison group, if they examined preterm or low birth weight infants, and reported primary data that could be used to measure the association between exposure to chorioamnionitis and the development of BPD. Studies classified chorioamnionitis as either clinical, histological, or microbiological. BPD was classified in the different studies as either (1) “Northway”: X-ray abnormalities persisting beyond one month of age in patients who continued to require oxygen or respiratory support; (2) “Bancalari”: need for oxygen on 28 of the first 28 days of life together with a compatible chest radiograph (or need for oxygen at 28 days of age, i.e., the National Institutes of Health (NIH) consensus definition of mild BPD); (3) “modern”: need for additional oxygen at 36 weeks postmenstrual age (i.e., the NIH consensus definition of moderate or severe BPD). The evaluated studies included randomised trials, prospective cohort, retrospective cohort, and case control studies. The meta-analysis of unadjusted (OR 1.89) and adjusted (OR1.58) showed a significant association between chorioamnionitis and BPD. The limitations of this analyses were that (1) the comparison groups within the studies were other preterm infants likely to be affected by vascular and other placental pathologies, also associated with perinatal inflammation and neonatal morbidity such as BPD [56, 57]; (2) gestational age and birthweight were confounding factors; (3) there was substantial heterogeneity across studies in the magnitude of the association between chorioamnionitis and BPD, and whether or not the association was statistically significant; (4) the heterogeneity remained after grouping studies by type of chorioamnionitis, definition of BPD, and whether the study included only very low birth weight infants or all preterm infants; (5) events such as exposure to antenatal steroids, postnatal sepsis, administration of surfactant, mode of ventilation, and patent ductus arteriosus were not known to be potential confounding factors at the time many of the studies were undertaken and therefore were not recorded; (6) the definition of chorioamnionitis was not consistent across the studies, and for instance, recent studies suggest that markers of fetal inflammatory response (leukocytosis or funisitis) are associated with the development of BPD, whereas chorioamnionitis alone is not [58, 59]; (7) the impact of ethnicity and the genetic background on the risk of BPD cannot be underestimated [36]; (8) the authors also found significant publication bias, and after controlling for publication bias for the adjusted data, the result was no longer significant; (9) also, the studies where the primary objective was not about the association between chorioamnionitis versus BPD showed no significant association, whereas studies reporting chorioamnionitis versus BPD as the primary objective were more likely to report a significant association; (10) the authors were restricted to data presented in the published reports and did not always have detailed information to control for important confounders, for instance, the gestational age; (11) the authors had to rely on the adjusted analyses as presented in the published reports and the variables which they included, which were not consistent across studies.

A study performed at our unit in order to assess the association between histological chorioamnionitis and BPD revealed a significant unadjusted association (OR 2.45 (95% CI: 1.16–5.18)), but when adjusted for gestational age and birthweight the association was no longer significant (OR 1.2 (95% CI: 0.51–2.95)) [60].

Been and Zimmermann[11] also reviewed the evidence of the association between intrauterine inflammation and pulmonary outcome. The authors also found numerous factors implicated in the interpretation of the results and that may explain, at least, part of the paradoxical findings among different studies. Inclusion criteria differ greatly among studies. Gestational age and birthweight differences were likely to affect results in some studies. Some studies selected their cohort by including only ventilated infants, or patients with premature prolonged rupture of membranes (PPROM), preterm labour, and suspected clinical chorioamnionitis. In contrast, cases of clinical chorioamnionitis or suspected maternal infection were excluded by others. Histopathological criteria and grading systems for diagnosing chorioamnionitis or fetal inflammation differ importantly between studies. The different definition of BPD complicates comparison of outcomes. Only a limited number of studies have adjusted the association for gestational age and birth weight. Some studies have performed multivariate models for other confounding factors without mentioning them, a fact that further complicates comparison of data. 

Small for gestational age and intrauterine growth restriction are fetal conditions that increase the risk of BPD and prolong the duration of mechanical ventilation in preterm infants younger than 32 weeks of gestation [61–63]. The main mechanisms to explain how intrauterine growth restriction modulates the risk of a BPD have been described in animals. In experimentally growth restricted preterm lambs, impaired growth of terminal airways, gas exchange units, and blood vessels have been shown [64, 65]. Furthermore, growth restriction leads to reduced expression of surfactant protein mRNA and could induce increase inflammatory activation [64]. In a study performed at our center, we found a different profile of cytokines in venous cord blood of small for gestational age preterm newborns and the association of low levels of venous cord blood IL-6 and IL-10 and moderate/severe BPD in small for gestational age preterm newborns [66]. 

Racial differences in chorioamnionitis prevalence and the pulmonary response to intrauterine inflammation may account for additional inconsistencies between studies, as chorioamnionitis and vaginal bacterial colonisation are more prevalent in non-White [46, 67, 68], while White race has a higher prevalence of BPD [46, 69]. Another factor is that over the time period the cohort study data have been collected, important changes in general practice in neonatal intensive care have taken place. The widespread implementation of antenatal steroids is the most obvious and probably the most influential example [70]. Other practice changes include the increased use of exogenous surfactant [71] and noninvasive modes of ventilatory support such as continuous positive airway pressure (CPAP) [72]. These may reduce secondary lung injury in chorioamnionitis-exposed infants and partly account for the apparently diminishing association between chorioamnionitis and BPD over time [73].

Probably the most informative study concerning indirect evidence of a link between chorioamnionitis and BPD is that reported by Van Marter and colleagues [73]. In a case-control design, very low birth weight infants with BPD were matched with infants without BPD on gestational age, birth weight, and hospital specific treatment strategies. In these infants chorioamnionitis was associated with a decreased risk for BPD if infants were ventilated for less than seven days. However, when infants were exposed to mechanical ventilation for more than seven days or had sepsis, chorioamnionitis significantly increased BPD risk, the effect being the most prominent with all three risk factors present. This suggests that while antenatal exposure to inflammation in itself may reduce BPD risk, it increases the susceptibility of the lung to postnatal injurious events.

## 6. Chorioamnionitis and Long-Term Respiratory Outcome

The association between chorioamnionitis and pulmonary outcome beyond the neonatal period has not been extensively studied. No significant differences in the use of supplemental oxygen, bronchodilators, and systemic or inhaled steroids were reported between patients with and without chorioamnionitis at 18–22 months' corrected age in the hydrocortisone trial, irrespective of neonatal hydrocortisone treatment [74]. In the study of Kumar and colleagues [75], chorioamnionitis and prematurity were shown to have a joint predisposing effect on recurrent wheezing and physician-diagnosed asthma at a mean age of 2.2 years, mainly in African-American children. Further investigation of the long-term effect of chorioamnionitis on respiratory outcome is warranted.

## 7. Conclusions

There is a strong evidence that histologic chorioamnionitis is associated with a reduction of incidence and severity of RDS. Short-term maturational effects on the lungs of extremely premature infants seem to be, however, accompanied by a greater susceptibility of the lung, eventually contributing to an increased risk of BPD. Genetic susceptibility to BPD is an evolving area of research and several studies have directly related the risk of BPD to genomic variants. There is a substantial heterogeneity across the studies in the magnitude of the association between chorioamnionitis and BPD, and whether or not the association is statistically significant. Considerable variation is largely depending on differences of inclusion and exclusion criteria, as well as on clinical and histopathological definitions. The presence of significant publication bias may exaggerate the magnitude of the association. Controlling for publication bias may conduct to adjusted results that are no longer significant. Recent studies generally seem to confirm the effect of chorioamnionitis on RDS incidence, while no effect on BPD is seen. 

Additional research is needed to explore the effect of antenatal inflammation on the early and late pulmonary outcome of the extreme premature newborn.
